# Nosocomial Infections in Non-COVID-19 Pediatric Patients Prior to and During the Pandemic in a Pediatric Intensive Care Unit

**DOI:** 10.7759/cureus.21451

**Published:** 2022-01-20

**Authors:** Sevcan İpek, Ahmet Şahin, Sukru Gungor, Sadık Yurttutan, Ufuk U Güllü, Sermin Inal, Şeyma Demiray

**Affiliations:** 1 Department of Pediatric Critical Care, Faculty of Medicine, Kahramanmaraş Sütçü İmam University, Kahramanmaraş, TUR; 2 Department of Infectious Diseases and Clinical Microbiology, Faculty of Medicine, Kahramanmaraş Sütçü İmam University, Kahramanmaraş, TUR; 3 Department of Pediatric Gastroenterology, Faculty of Medicine, Kahramanmaraş Sütçü İmam University, Kahramanmaraş, TUR; 4 Department of Neonatalogy, Faculty of Medicine, Kahramanmaraş Sütçü İmam University, Kahramanmaraş, TUR; 5 Department of Pediatric Cardiology, Faculty of Medicine, Kahramanmaraş Sütçü İmam University, Kahramanmaraş, TUR; 6 Infection Control Committee: Nursing, Faculty of Medicine, Kahramanmaraş Sütçü İmam University, Kahramanmaraş, TUR; 7 Department of Pediatrics, Faculty of Medicine, Kahramanmaraş Sütçü İmam University, Kahramanmaraş, TUR

**Keywords:** pediatric intensive care unit, pandemic, nosocomial infections, covid-19, children

## Abstract

Background: Nosocomial infections are a global threat to human health worldwide.

Aim: This study aimed to investigate the change of nosocomial infection factors in equivalent historical periods in pediatric patients without COVID-19 before and during the pandemic in the pediatric intensive care unit.

Method: The study was planned retrospectively. Data on hospital infection rates, incidence densities, invasive device-associated infections, infectious agents, comorbid diseases, and invasive procedures in non-COVID-19 pediatric patients were obtained from the medical records for the periods of April-September 2019 and April-September 2020 in the pediatric intensive care unit. Hand hygiene compliance rates of healthcare workers were evaluated.

Results: Prior to the pandemic, the number of patients was 332, comprising 2,377 patient days with a nosocomial infection rate of 5.12, and an incidence density of 7.15. During the pandemic, the number of patients was 221, comprising 2,260 patient days with a nosocomial infection rate of 4.52, and incidence density of 4.43. Prior to the pandemic, there were 28.80% cases of *Klebsiella pneumoniae*, 23.81% of *Pseudomonas aeruginosa,* 9.52% of *Enterococcus faecium,* and 4.76% of *Enterococcus faecalis.* During the pandemic, there were decreased 14.29% cases of *Klebsiella pneumoniae* while *Pseudomonas aeruginosa, Enterococcus faecium, and Enterococcus faecalis* was not seen. Prior to the pandemic, the hand hygiene compliance rate was 94.83%, and during the pandemic, it was found to be 99.44%.

Conclusion: This study showed that the spread of bacteria such as *Klebsiella pneumoniae, Pseudomonas aeruginosa, *vancomycin-resistant enterococci, and *Stenotrophomonas maltophilia,* which are a major public health threat, can be decreased by applying simple standard methods.

## Introduction

Nosocomial infections (NIs) are common in pediatric intensive care units (PICUs), due to the poor clinical condition of patients, increased invasive procedures, low immune response, prolonged hospitalization, and frequent use of antibiotics [1]. One study showed that 19.1% of patients hospitalized in ICUs had at least one healthcare-associated infection [2]. In a prevalence study conducted in Europe, the rate of NI in PICUs was reported to be 23.6% [3]. In our country, it ranges from 9.1% to 37% in PICUs [4]. NIs are associated with high costs of health care and increased morbidity and mortality, with a reported mortality rate of over 50% in some studies [5,6].

The Chinese Government announced to the world in December 2019 that a new type of coronavirus (SARS CoV-2) was isolated [7]. With this rapidly growing and uncontrolled pandemic, the health systems of different countries have shown different responses in surveillance, diagnosis, and treatment, and it has been reported that not all populations are equally affected in terms of the number of cases, serious illness, and death [8]. SARS CoV-2 is transmitted from person to person through droplets, contact, and in some cases aerosol. Therefore, patients with suspected COVID-19 and healthcare professionals must comply with standard, droplet and contact isolation precautions to prevent transmission [9].

During the COVID-19 pandemic, we observed that healthcare workers, who are at the highest risk of transmission, adapted more to standard isolation measures than prior to the pandemic, and that there was an increase in behaviors such as hand washing, and the use of gloves, masks, and glasses. We found it worthwhile to study the changes in other infectious agents in the PICU during the pandemic process, as it may shed light on the use of antibiotics and infection protection control measures. In this study, we examined the change of NI factors at equivalent historical cross-sections prior to and during the pandemic in a PICU in non-COVID-19 pediatric patients.

## Materials and methods

Study design

The study was planned retrospectively. This study was carried out in a PICU of a tertiary health institution in a university hospital. The study was conducted with consent from the local ethics committee (2020/17-13). The hospital has 120 adults, 10 PICU, and 36 neonatal intensive care beds. In the PICU, there are 10 intensive care beds (two of which are in isolated rooms) in an area separate from the pediatric clinic. The first SARS-CoV2 case in Turkey was reported on March 11, 2020. Data on NIs were obtained from the medical records at the non-COVID-19 PICU for the periods of April-September 2019 and April-September 2020.

Data collection

Patients staying less than 48 hours in the PICU and patients with an infection that had developed more than 48 hours before admission were excluded from the study. The nurses of the Infection Control Committee and the Department of Infectious Diseases conduct daily visits to the PICU. It is hospital policy that employees in the PICU are obliged to use scrubs and surgical masks. Peripheral blood culture, tracheal aspirate culture, urine culture, and when available catheter culture, catheter tip cultures when catheters are removed, and wound site culture samples were taken from all patients whose body temperature was above 38°C. Hematological and biochemical parameters were examined. Radiological examinations were made when necessary. Considering the physical examination findings and the clinical condition of the patients, the results were evaluated together. NIs were diagnosed using the Centers for Disease Control and Prevention criteria [10]. The notes formed as a result of daily visits were retrospectively analyzed.

The rate of NI was calculated by using the number of infections detected in the PICU/number of inpatients in PICU x 100 formula. Incidence density was calculated by using the number of infections in PICU/total hospitalization days of patients in PICU x 1,000. Ventilator-associated pneumonia (VAP) rate was calculated by using the VAP number/ventilator days x 100 formula. The catheter-associated urinary tract infection (CI-UTI) rate was calculated by using the CI-UTI number/urinary catheter days x 100. The central-line-associated bloodstream infections (CLABSI) rate was calculated by using the CLABSI number/central venous catheterization days x 100 formula. 

Variables

In this study, the demographic data of the patients, the use of proton-pump inhibitors during infection, the use of steroids, 30-day mortality after infection, follow-up in intensive care, invasive device-related infections, antibiotic general resistance rates, hand hygiene compliance rates, infection types, infection factors, and infection rates were evaluated.

Statistical analysis

SPSS version 25.0 statistical package program (IBM Corp, Armonk, NY) was used in the analysis of the data [11]. The conformity of the obtained data to normal distribution was examined by visual (histogram and probability graphics) and analytical methods (Kolmogorov-Smirnov/Shapiro-Wilk tests). In the analysis of the descriptive statistics of the study, mean ± standard deviation (SD) for continuous numerical variables, minimum-maximum values, number (n), and percentage (%) for categorical variables were used. Chi-square or Fisher's test was used for comparison of categorical variables in the comparison of groups. For the comparison of continuous variables, an independent-sample t-test or Mann-Whitney U-test was used. p <0.05 was accepted as the statistical significance limit value.

## Results

A total of 553 patients were included in the study. Forty-eight percent (267) of them were male and 52% (286) were female. The mean age was 128 months (min-max: 1-216). Prior to the pandemic, the number of patients was 332, comprising 2,377 patient days, 17 infections, with a rate of 5.12, and an incidence density of 7.15. During the pandemic, the number of patients was 221, comprising 2,260 patient days, 10 infections, with a rate of 4.52, and an incidence density of 4.43 (Table 1).

**Table 1:** Non-COVID-19 PICU infection rates and proper hand hygiene prior to and during the COVID-19 pandemic PICU, pediatric intensive care unit

Variable	2019	2020
Infection rate		
Patient count	332	221
Inpatient days	2,377	2,260
Infection count	17	10
Rate	5.12	4.52
Density	7.15	4.43
Hand hygiene compliance		
Appropriate observation count	110	179
Total observation count	116	180
Compliance rate (%)	94.83	99.44

There were 17 NI attacks prior to the pandemic and 10 during the pandemic. The growth of two microorganisms in a single culture was detected in eight cultures taken prior to and during the pandemic. Twenty-one agents prior to the pandemic and 14 agents during the pandemic were observed. A total of 27 NI attacks were detected in 20 patients (Table 2).

**Table 2:** Non-COVID-19 PICU infections and antibiotic resistance prior to and during the COVID-19 pandemic CLABSI, central-line-associated bloodstream infections; VAP, ventilator-associated pneumonia; ESBL, extended-spectrum β-lactamase; PICU, pediatric intensive care unit

Variable	2019	2020
CLABSI		
Count	8 (47%)	5 (50%)
Rate	2.41	2.26
Density	3.37	2.21
VAP		
Count	9 (53%)	2 (20%)
Rate	2.71	0.9
Density	3.79	0.44
Meningitis/ventriculitis		
Count	-	1 (10%)
Rate	-	0.45
Density	-	0.44
Decubitus ulcer		
Count	-	1 (10%)
Rate	-	0.45
Density	-	0.44
Clinically defined pneumonia		
Count	-	1 (10%)
Rate	-	0.45
Density	-	0.44
Resistance: ESBL		
*Klebsiella pneumoniae* (%)	83.33	50
Resistance: KARBAPENEM		
*Acinetobacter baumannii* (%)	100	100
*Klebsiella pneumoniae* (%)	66.67	50
*Pseudomonas aeruginosa* (%)	60	-
Resistance: KOLISTIN		
*Acinetobacter baumannii* (%)	0	16.67
Resistance: VRE		
*Enterococcus faecalis* (%)	100	-
*Enterococcus faecium* (%)	100	-

The demographic characteristics of the patients are presented in Table 3.

**Table 3:** Characteristics of patients who developed infection in non-COVID-19 PICU before and during COVID-19 pandemic ^a^Fisher's exact, ^b^Mann-Whitney U test, ^c^Chi-square test TPN: total parenteral nutrition

Variable	2019	2020	p
Sex (female/male)	7/5	3/5	0.65^a^
Age (months) median (min-max)	16.5 (3-178)	35.5 (10-152)	0.23^b^
Weight (kg) median (min-max)	7.4 (2.4-42)	15.5 (6.5-50)	0.9^b^
30-day mortality	5 (29.4%)	2(20%)	0.67^a^
Length of hospital stay (days) median (min-max)	120 (19-343)	75 (23-168)	0.48^b^
Proton-pump inhibitor	14 (82.4%)	7 (87.5%)	1^a^
Steroid	4 (23.5)	0	0.26^a^
TPN	3 (17.6%)	0	0.27^a^
Central venous catheter and port	15 (88.2%)	6 (60%) and port 3(30%)	0.56^c^

The 30-day mortality after infection was 29.4% prior to the pandemic and 20% during the pandemic (p = 0.68). The average length of stay in the hospital was 120 days (19-343) and 75 (23-168) days, respectively, prior to and during the pandemic (p = 0.48). While proton-pump inhibitor was used in 14 patients (82.4%) in infections detected before the pandemic, it was found that it was used in nine patients (87.5%) during the pandemic (p =0.7). While steroid use was 4 (23.5%) prior to the pandemic, it was found that the patients did not receive any steroids during the pandemic. There was no statistical difference in terms of steroid use (p = 0.26). The use of central venous catheters prior to the pandemic was seen in 15 patients (88.2%), during the pandemic the use of central venous catheters was seen in six patients (60%) and the use of ports in three patients (30%) was recorded. There was no statistically significant difference between the groups (p = 0.56). While there were three patients (17.6%) prior to the pandemic in terms of the use of total parenteral nutrition (TPN) during infection, it was determined that there were no patients who received it during the pandemic. This was not statistically different (p = 0.27) (Table 2).

Prior to the pandemic, NIs were 8 (47%) with CLABSI, its rate was 2.41, its density was 3.37, and the number of VAP was 9 (53%), its rate was 2.71, and its density was 3.79. During the pandemic, the number of meningitis/ventriculitis was 1 (10%), with a rate of 0.45 and a density of 0.44. Additionally, the decubitus ulcer infection number was 1 (10%), with a rate of 0.45 and a density of 0.44. The CLABSI number was 5 (50%), with a rate of 2.26 and a density of 2.21. The clinically defined pneumonia number was 1 (10%), with a rate of 0.45 and a density of 0.44. The VAP number was 2 (20%), with a rate of 0.9 and a density of 0.88 (Table 1).

**Figure 1:**
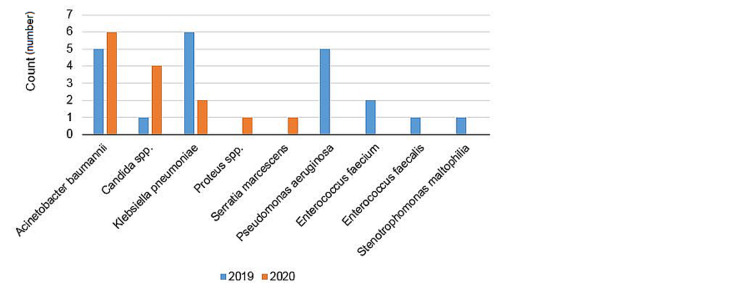
Nosocomial infection agents (count) prior to and during the COVID-19 pandemic.

**Figure 2:**
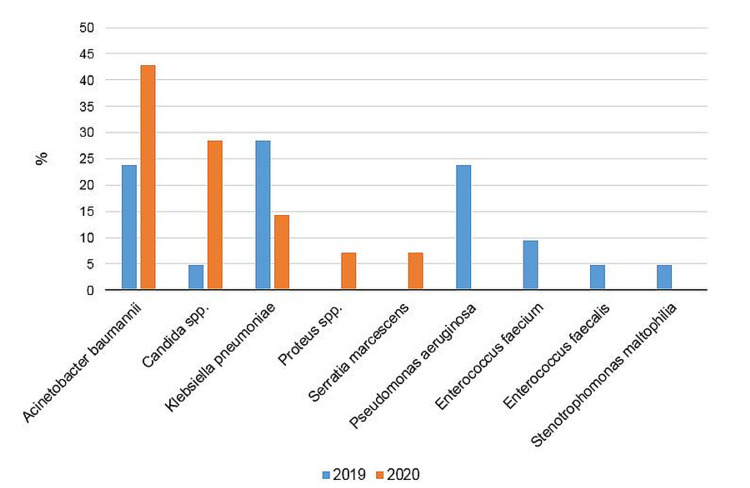
Nosocomial infection agents (%) prior to and during the COVID-19 pandemic.

Infection agents seen prior to and during the pandemic were as follows: Prior to the pandemic, *Acinetobacter baumannii* - 5 cases (23.81%), *Klebsiella pneumoniae* - 6 cases (28.8%), *Pseudomonas aeruginosa* - 5 cases (23.81%), *Enterococcus faecium* - 2 cases (9.52%), *Enterococcus faecalis* - 1 case (4.76%), *Stenotrophomonas maltophilia* - 1 case (4.76%), and *Candida* spp. - 1 case (4.76%) were seen. During the pandemic, *Klebsiella pneumoniae* - 2 cases (14.29%), *Acinetobacter baumannii* - 6 cases (42.86%), *Proteus* spp. - 1 case (7.14%), *Serratia marcescens* - 1 case (7.14%), and *Candida parapsilosis* - 4 cases (28.57%) were seen (Figures 1, 2).

 

The general resistance rates to antibiotics prior to the pandemic are shown in Table 1. 

Prior to the pandemic, a total of 116 hand hygiene observations were made, of which 110 were found to have been appropriate. The pre-pandemic hand hygiene compliance rate was 94.83%. A total of 180 hand hygiene observations were made during the pandemic, of which 179 were reported to have been appropriate. The pandemic hand hygiene compliance rate was found to be 99.44% (Table 1).

## Discussion

In this study, the epidemiology of NI in our PICU prior to and during the pandemic was investigated. Compared to the pre-pandemic period, the hospital infection rate and incidence density decreased during the pandemic (5.12 vs. 4.52).

*Klebsiella pneumoniae* from the Enterobacteriaceae family is an opportunistic Gram-negative pathogen that causes pneumonia, blood-spreading infections, and urinary tract infections. The gastrointestinal tract of patients and the hands of hospital staff are the main reserves and can lead to nosocomial outbreaks [12]. The problem of cephalosporin-resistant *Klebsiella pneumoniae* initially linked to the production of broad-spectrum β-lactamases is now further complicated by the emergence of strains resistant to carbapenems [13]. *Klebsiella pneumoniae* is currently the most common strain among carbapenem-resistant Enterobacteriaceae, and the emergence of carbapenem-resistant *Klebsiella pneumoniae* has been declared an 'urgent and critical' threat by national and international organizations [14]. In our study, a significant decrease was shown in the prevalence of *Klebsiella pneumoniae* and antimicrobial resistance during the pandemic. Compliance of healthcare professionals with isolation measures has an important role in this decrease.

*Stenotrophomonas maltophilia* is a Gram-negative bacillus commonly found in hospitals. Intrinsically, they are resistant to beta lactam antibiotics and they show nearly 100% resistance to carbapenem group drugs. In addition, because of their resistance to aminoglycosides, they cause difficulties in the management of patients whose immune systems are suppressed. Inappropriate antibiotic use, the use of immunosuppressant drugs, and invasive medical devices have led to an increase in the rate of NIs caused by *Stenotrophomonas maltophilia* [15]. In our study, it was determined that *Stenotrophomonas maltophilia* was seen in one case prior to the pandemic, but no *Stenotrophomonas maltophilia* was observed during the pandemic. We think that the contact measures, which the staff started to implement strictly to protect themselves during the pandemic, had an effect.

*Pseudomonas aeruginosa* is a Gram-negative, ubiquitous opportunistic pathogen. It can lead to life-threatening acute and chronic infections, especially in immunocompromised patients and patients with cystic fibrosis [16]. While it was observed with a rate of 23.81% prior to the pandemic in this study, no *Pseudomonas* infection was detected during the pandemic. This is very meaningful in terms of showing us how important it is to comply with standard precautions in preventing the spread of infections.

Enterococci are facultative Gram-positive cocci, with *Enterococcus faecium* and *Enterococcus faecalis* causing human infections. These organisms are a normal member of the gastrointestinal flora and are opportunistic pathogens in immunocompromised and critically ill patients. They can cause skin and soft tissue infections, urinary tract infections, infective endocarditis, and invasive device-related infections, and lead to nosocomial outbreaks [17]. Prior to the pandemic, 14.28% vancomycin-resistant enterococcus (VRE) was observed, while enterococcal infections were not observed during the pandemic. Although the percentage of central venous catheter use did not change, we associate this decrease with the willingness of the staff to comply with contact isolation measures.

*Acinetobacter baumannii*, another Gram-negative agent, has increased during the pandemic compared to the previous period. It is an opportunistic pathogen that usually infects patients in intensive care units. It is seen as a global public health threat due to its ability to survive for months in dry environments, to be resistant to disinfectants, to form biofilms, and to develop multi-antibiotic resistance [18,19]. In our study, when we examined those patients who reproduced *Acinetobacter baumannii* in detail to understand what caused the increase during the pandemic, it was found that the first *Acinetobacter baumannii-*breeding patient of the year was taken to external ventricular drainage in the operating room and then it was produced in the blood culture taken. In another patient with *Acinetobacter baumannii* growth, a gastrostomy was done in the operating room and then it was found that *Acinetobacter baumannii* developed in two more patients. We confirmed that there was an *Acinetobacter baumannii* epidemic in the operating room at the same time. It was discovered that another patient who had *Acinetobacter baumannii* was transferred from another service. The reason for the increase in the prevalence of *Acinetobacter baumannii* was thought to be related to patients in the operating room. However, we would expect a decrease in *Acinetobacter baumannii* infection, as the incidence of *Klebsiella pneumoniae* was reduced and VRE was not seen at all, with the meticulous implementation of the standard precautions we saw in our staff. We speculate that standard measures may be insufficient to prevent the spread of *Acinetobacter baumannii*, and therefore, additional emergency preventions should be sought.

*Serratia marcescens* is a Gram-negative aerobic organism, which is common in hospital settings and can cause hospital outbreaks. These outbreaks are difficult to deal with, and they can last for months to years [20]. *Proteus* spp. belongs to the family of Enterobacteriaceae found in the gastrointestinal tract, and it is widely found in water and soil. It is intrinsically resistant to colistin and causes NIs [21]. In our study, we were surprised that *Serratia marcescens* and *Proteus* spp., which were not seen prior to the pandemic, were detected in two separate cases in intensive care during the pandemic as there was a marked decrease in the incidence of other Gram-negative bacteria, with some never seen. When the patients with *Serratia marcescens* and *Proteus* spp. were examined retrospectively, it was found that the two patients had undergone surgical procedures in the operating room and it was thought that these infections were related to this. These infections did not spread later in the ICU and remained as single cases. 

In this study, an increase was found in fungal infections during the pandemic compared to the period prior to the pandemic. Candida spp. are important pathogens worldwide in opportunistic fungal infections, causing serious NIs, and candidiasis is mostly caused by the endogenous spread of the patient's microflora. Risk factors for invasive candidiasis include long-term hospitalizations in ICUs, invasive procedures, the use of broad-spectrum antibiotics for multi-drug-resistant bacteria, the use of TPN, and gastrointestinal surgery [22-24]. When patients who developed invasive candidiasis during the pandemic were examined in depth, it was found that both ventriculoperitoneal shunt and gastrostomy were present in one patient and ventriculoperitoneal shunt in two patients. It was found that the patient who developed invasive candidiasis prior to the pandemic had repeatedly undergone gastrointestinal surgery due to short bowel syndrome.

In our study, 99.44% compliance with hand hygiene was observed during the pandemic. Although compliance with hand hygiene is the most effective and cheapest method in reducing the rates of NIs, unfortunately, compliance with hand hygiene in healthcare workers has been reported at a very low level in some studies [25]. Length of hospital stay, use of proton-pump inhibitors, steroids, and TPN facilitate the development of NI [26,27]. In the study, there was no difference between the length of hospital stay, proton-pump inhibitor use, steroid use, and TPN intake prior to and during the COVID-19 pandemic. Therefore, with the increase in hand hygiene compliance with the pandemic, a significant decrease in NIs has been detected in our clinic.

The strengths of this study can be listed as the multidisciplinary execution of infection control practices, the long-term collaboration of the infection control team, the use of advanced molecular tests, and the high level of hospital management support. In summary, it is important to carry out infection control practices as a whole. All relevant units must work together continuously. Infection control measures in our hospital are directly or indirectly related to each other. By carrying out all infection control measures together with management support, healthcare-associated infection rates in an endemic country in terms of pathogens that have developed multi-drug resistance can be reduced to levels much lower than in the developed countries. The limitations of our study are that it was a single-center study, it was performed in a 10-bed PICU, and the number of beds and the number of patients were low. In addition, the fact that it is not prospective is an important factor limiting the study. 

## Conclusions

In this study, we investigated the change of NIs in pediatric patients without COVID-19 before and during the pandemic in PICU. Compliance with hand hygiene was found to be as high as 99.44% in our clinic during the pandemic compared to before. During the pandemic, a significant decrease was detected in the rate of hospital infections that pose a threat to public health compared to that prior to the pandemic. Therefore, this study is important in terms of showing that the spread of bacteria such as *Klebsiella pneumoniae*, *Pseudomonas aeruginosa*, VRE, and *Stenotrophomonas maltophilia*, which are an important public health threat, can be decreased by applying simple standard methods, and it is understood that increased training should be continued even when the pandemic is over.
